# 3-[(4-Phen­oxy­phen­yl)sulfan­yl]-5-phenyl-1*H*-1,2,4-triazole

**DOI:** 10.1107/S1600536814008204

**Published:** 2014-04-30

**Authors:** Raja Ben Othman, Mathieu Marchivie, Franck Suzenet, Sylvain Routier

**Affiliations:** a École Supérieure des Sciences et de Technologie de Hammam Sousse (ESST), Rue Lamine Abassi 4011 Hammam Sousse, Laboratoire d’Application de la Chimie aux Ressources et Substances Naturelles et l’Environnement (LACReSNE), Faculté des Sciences de Bizerte, 7021 Zarzouna, Bizerte, Tunisia; b Institut de Chimie Organique et Analytique, Université d’Orléans, UMR CNRS 7311, BP 6759, 45067 Orléans Cedex 2, France; c ICMCB CNRS UPR 9048, Université de Bordeaux, 87 Avenue du Docteur Schweitzer, 33608 Pessac Cedex, France

## Abstract

The title compound, C_20_H_15_N_3_OS, is V-shaped. In the 4-phen­oxy­phenyl group, the two rings are inclined to one another by 74.52 (13)°. These rings are inclined to the triazole ring by 72.20 (15) and 72.30 (15)°, respectively. The phenyl ring is inclined to the triazole ring by 10.85 (12)°. In the crystal, mol­ecules are linked *via* N—H⋯N hydrogen bonds, forming chains propagating along [010]. These chains are linked *via* pairs of C—H⋯S hydrogen bonds, forming sheets lying parallel to the *ac* plane.

## Related literature   

For the synthesis, properties and various biological activities of functionalizated 1,2,4-triazole derivatives, see: Holla *et al.* (2002 ▶, 2003 ▶); Walczak *et al.* (2004 ▶); Zitouni *et al.* (2005 ▶); Prasad *et al.* (2009 ▶); Wael *et al.* (2012 ▶); Almasirad *et al.* (2004 ▶); Amir & Shikha (2004 ▶); Kane *et al.* (1988 ▶); Akhtar *et al.* (2010 ▶). For the crystal structures of related *N*-free triazole derivatives, see for example: Qadeer *et al.* (2007 ▶); and for *N*-subsituted derivatives, see for example: Zhao *et al.* (2010 ▶); Wu *et al.* (2009 ▶). Working with sulfur-containing heterocycles may provide unexpected results and the title compound was obtained within an unprecedented series of results, see: Ben Othman *et al.* (2014 ▶).
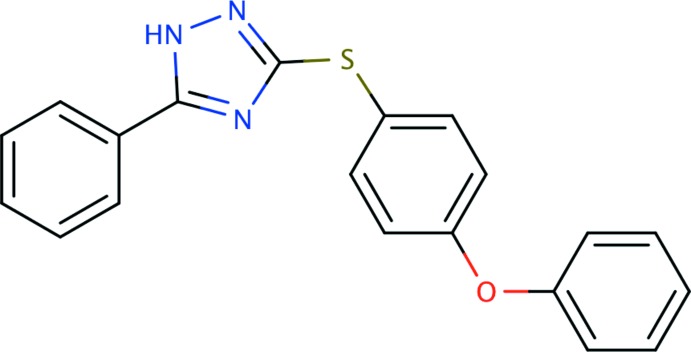


## Experimental   

### 

#### Crystal data  

C_20_H_15_N_3_OS
*M* *_r_* = 345.41
Monoclinic, 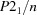

*a* = 16.6112 (12) Å
*b* = 5.8445 (5) Å
*c* = 17.5415 (10) Å
β = 93.131 (5)°
*V* = 1700.5 (2) Å^3^
*Z* = 4
Mo *K*α radiation
μ = 0.20 mm^−1^
*T* = 293 K
0.35 × 0.25 × 0.12 mm

#### Data collection  

Bruker–Nonius KappaCCD diffractometer
Absorption correction: multi-scan (*SADABS*; Sheldrick, 1996 ▶) *T*_min_ = 0.932, *T*_max_ = 0.976
44120 measured reflections
3099 independent reflections
2333 reflections with *I* > 2σ(*I*)
*R*_int_ = 0.034

#### Refinement  

*R*[*F* ^2^ > 2σ(*F* ^2^)] = 0.052
*wR*(*F* ^2^) = 0.152
*S* = 1.02
3099 reflections
207 parameters
H atoms treated by a mixture of independent and constrained refinement
Δρ_max_ = 0.28 e Å^−3^
Δρ_min_ = −0.30 e Å^−3^

### 

Data collection: *COLLECT* (Bruker–Nonius, 1998) ▶; cell refinement: *DIRAX/LSQ* (Duisenberg, 1992 ▶); data reduction: *EVALCCD* (Duisenberg *et al.*, 2003 ▶); program(s) used to solve structure: *SHELXS97* (Sheldrick, 2008 ▶); program(s) used to refine structure: *SHELXL97* (Sheldrick, 2008 ▶); molecular graphics: *OLEX2* (Dolomanov *et al.*, 2009 ▶); software used to prepare material for publication: *OLEX2*.

## Supplementary Material

Crystal structure: contains datablock(s) I. DOI: 10.1107/S1600536814008204/su2718sup1.cif

Structure factors: contains datablock(s) I. DOI: 10.1107/S1600536814008204/su2718Isup2.hkl

Supporting information file. DOI: 10.1107/S1600536814008204/su2718Isup3.cml

CCDC reference: 991904

Additional supporting information:  crystallographic information; 3D view; checkCIF report

## Figures and Tables

**Table 1:** Hydrogen-bond geometry (Å, °)

*D*—H⋯*A*	*D*—H	H⋯*A*	*D*⋯*A*	*D*—H⋯*A*
N3—H3⋯N2^i^	0.91 (3)	2.05 (3)	2.944 (3)	170 (2)
C16—H16⋯S1^ii^	0.93	2.77	3.694 (2)	170

Symmetry codes: (i) 
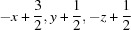
; (ii) 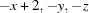.

**Fig. 1.**
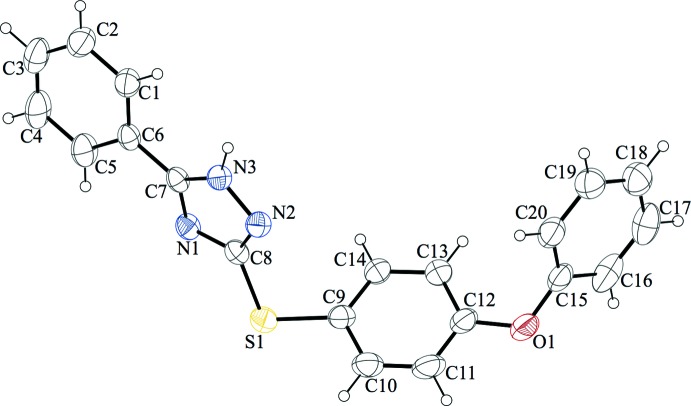
The molecular structure of the title molecule, with atom labelling. Displacement ellipsoids are drawn at the 30% probability level.

**Fig. 2.**
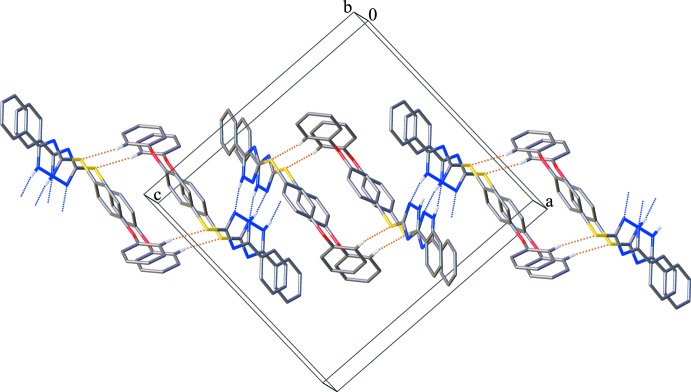
A perspective view along the b axis of the crystal pack of the title compound. Hydrogen bonds are shown as dashed lines (see Table 1 for details; H atoms not involved in hydrogen bonding have been omitted for calrity).

**Fig. 3.**
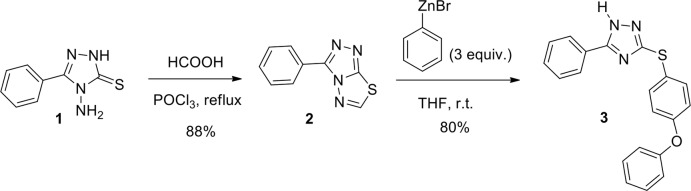
The synthetic route of the title compound.

## supplementary crystallographic information

### 1. Comment

From a medicinal chemistry point of view, it is of great interest to develop efficient methods for the synthesis and the functionalization of 1,2,4-triazoles, as they are known to possess a wide range of biological activities, such as, as anticancer (Holla *et al.*, 2002, 2003) antitubercular (Walczak *et al.*, 2004), antimicrobial (Zitouni *et al.*, 2005; Prasad *et al.*, 2009; Wael *et al.*, 2012), anticonvulsant (Almasirad *et al.*, 2004), anti-inflammatory, analgesic (Amir & Shikha, 2004), antidepressant (Kane *et al.*, 1988), and urease inhibitors (Akhtar *et al.*, 2010). Thus, the synthesis of 1,2,4-triazoles and the investigation of their chemical and biological behaviour have acquired more importance in recent decades for these reasons.

An efficient and convenient method was developed for the formation of substituted thiotriazoles via an organometallic addition and subsequent ring opening sequence of 3-substituted-[1,2,4]triazolo[3,4-][1,3,4]thiadiazole. This method is applicable to a wide range of substrates containing different functional groups and furnishes excellent yields of the corresponding unsubstituted 3 or 5-alkyl, aryl, alkynyl and alkenyl sulfanyl-1,2,4-triazole products.

Interestingly, working with sulfur-containing heterocycles may provide unexpected results and we report herein on the crystal structure of one derivative obtained within an unprecedented series of results (Ben Othman *et al.*, 2014).

The molecular structure of the title molecule is illustrated in Fig. 1. The molecule is V-shaped about atom S1. In the 4-phenoxyphenyl group the two rings (C9-C14 and C15-C20) are inclined to one another by 74.52 (13) °. These rings are inclined to the triazole ring (N1-N3/C7/C8) by 72.20 (15) and 72.30 (15) °, respectively. The phenyl ring (C1-C6) is inclined to the triazole ring by 10.85 (12) °.

In the crystal, molecules are linked via N-H···N hydrogen bonds forming chains propagating along [010]; see Table 1 and Fig. 2. These chains are linked via pairs of C-H···S hydrogen bonds forming sheets lying parallel to the ac plane (Table 1 and Fig. 2).

### 2. Experimental

For the synthesis of the title compound, see Fig. 3. In a 25 ml flask, phenyl ZnBr solution in THF (1.5 mmol, 0.5*M*) was added drop wise under argon at room temperature to a solution of 3-Phenyl-[1,2,4]triazolo[3,4-*b*][1,3,4]thiadiazole (0.5 mmol) in THF (5 ml), and the mixture was stirred for 25 min (see Fig 3). At the end of the reaction, the mixture was quenched with 15 mL of an aqueous solution of saturated NH_4_Cl, and extracted with CH_2_Cl_2_ (2 × 20 ml). The extract was dried over magnesium sulfate, filtered and concentrated *in vacuo*. The residue was purified by flash column chromatography on silica gel (eluent 6:4 petroleum ether/AcOEt). The title compound was obtained as a white solid in 80% yield. *R*_f_ = 0.60 (petroleum ether/EtOAc, 6:4); M.p. 318-320 K. HRMS (EI—MS): *m/z* calcd for C_20_H_15_N_3_OS: 346.10086 [*M* + H]^+^, found: 346.10112. Crystals of the title compound were obtained by vapor diffusion of petroleum ether into a solution of the title compound in a CH_2_Cl_2_/Et_2_O/pentane mixture. Spectroscopic data for the title compound is available in the archived CIF.

### 3. Refinement

The NH H atom was located in a difference Fourier map and freely refined. The C-bound H atoms were included in calculated positions and treated as riding atoms: C-H = 0.93 Å with U_iso_(H) = 1.2U_eq_(C).

### Crystal data

**Table:**

C_20_H_15_N_3_OS	*Z* = 4
*M**_r_* = 345.41	*F*(000) = 720
Monoclinic, *P*2_1_/*n*	*D*_x_ = 1.349 Mg m^−^^3^
Hall symbol: -P 2yn	Mo *K*α radiation, λ = 0.71073 Å
*a* = 16.6112 (12) Å	µ = 0.20 mm^−^^1^
*b* = 5.8445 (5) Å	*T* = 293 K
*c* = 17.5415 (10) Å	Block, colourless
β = 93.131 (5)°	0.35 × 0.25 × 0.12 mm
*V* = 1700.5 (2) Å^3^	

### Data collection

**Table:**

Bruker–Nonius KappaCCD diffractometer	3099 independent reflections
Radiation source: sealed X-ray tube	2333 reflections with *I* > 2σ(*I*)
Graphite monochromator	*R*_int_ = 0.034
profile data from φ scans and ω scans	θ_max_ = 25.4°, θ_min_ = 3.3°
Absorption correction: multi-scan (*SADABS*; Sheldrick, 1996)	*h* = −20→20
*T*_min_ = 0.932, *T*_max_ = 0.976	*k* = −7→6
44120 measured reflections	*l* = −21→21

### Refinement

**Table:**

Refinement on *F*^2^	Primary atom site location: structure-invariant direct methods
Least-squares matrix: full	Secondary atom site location: difference Fourier map
*R*[*F*^2^ > 2σ(*F*^2^)] = 0.052	Hydrogen site location: mixed
*wR*(*F*^2^) = 0.152	H atoms treated by a mixture of independent and constrained refinement
*S* = 1.02	*w* = 1/[σ^2^(*F*_o_^2^) + (0.0707*P*)^2^ + 1.0533*P*] where *P* = (*F*_o_^2^ + 2*F*_c_^2^)/3
3099 reflections	(Δ/σ)_max_ = 0.001
207 parameters	Δρ_max_ = 0.28 e Å^−^^3^
0 restraints	Δρ_min_ = −0.30 e Å^−^^3^

### Special details

**Table:**

Experimental. Spectroscopic data for the title compound: IR (ATR diamond): *ν* (cm^-1^) = 3082, 2927, 2864, 1581, 1482, 1324, 1242, 1006, 869, 786, 601, 725, 688; ^1^H NMR (400 MHz, CDCl_3_) *δ* (p.p.m.) = 12.92 (br. s, 1H), 7.90 (d, *J* = 6.9 Hz, 2H), 7.47 (d, *J* = 8.4 Hz, 2H), 7.43–7.28 (m, 5H), 7.15 (t, *J* = 7.3 Hz, 1H), 6.96 (d, *J* = 7.8 Hz, 2H), 6.84 (d, *J* = 8.4 Hz, 2H); ^13^C NMR DEPT (101 MHz, CDCl_3_): *δ* (p.p.m.) = 134.9 (2CH_Ar_), 130.2 (CH_Ar_), 129.9 (2CH_Ar_), 128.8 (2CH_Ar_), 126.5 (2CH_Ar_), 124.1 (CH_Ar_), 119.7 (2CH_Ar_), 119 (2CH_Ar_).
Geometry. All e.s.d.'s (except the e.s.d. in the dihedral angle between two l.s. planes) are estimated using the full covariance matrix. The cell e.s.d.'s are taken into account individually in the estimation of e.s.d.'s in distances, angles and torsion angles; correlations between e.s.d.'s in cell parameters are only used when they are defined by crystal symmetry. An approximate (isotropic) treatment of cell e.s.d.'s is used for estimating e.s.d.'s involving l.s. planes.

### Fractional atomic coordinates and isotropic or equivalent isotropic displacement parameters (Å^2^)

**Table:**

	*x*	*y*	*z*	*U*_iso_*/*U*_eq_	Occ. (<1)
S1	0.74490 (4)	0.10572 (13)	0.07643 (5)	0.0708 (3)	
N3	0.69265 (12)	0.6140 (4)	0.19811 (11)	0.0507 (5)	
N2	0.74402 (12)	0.4385 (4)	0.18506 (11)	0.0540 (5)	
N1	0.64442 (12)	0.4567 (4)	0.09308 (11)	0.0539 (5)	
C7	0.63379 (13)	0.6216 (4)	0.14328 (13)	0.0474 (5)	
C6	0.56826 (8)	0.7865 (3)	0.13936 (9)	0.0518 (6)	
C1	0.56878 (10)	0.9796 (3)	0.18563 (10)	0.0661 (7)	
H1	0.6109	1.0031	0.2219	0.079*	
C2	0.50635 (12)	1.1377 (3)	0.17765 (12)	0.0811 (9)	
H2	0.5067	1.2669	0.2086	0.097*	
C3	0.44340 (10)	1.1026 (4)	0.12341 (13)	0.0885 (11)	
H3A	0.4016	1.2083	0.1181	0.106*	
C4	0.44288 (9)	0.9094 (4)	0.07715 (11)	0.0903 (11)	
H4	0.4008	0.8859	0.0409	0.108*	
C5	0.50531 (11)	0.7514 (3)	0.08512 (10)	0.0746 (8)	
H5	0.5050	0.6222	0.0542	0.090*	
C9	0.85042 (16)	0.1109 (4)	0.09473 (14)	0.0575 (6)	
C12	1.01532 (19)	0.0906 (5)	0.1147 (2)	0.0795 (9)	
C8	0.71159 (14)	0.3513 (4)	0.12111 (13)	0.0508 (6)	
C14	0.89725 (16)	0.2919 (5)	0.07283 (17)	0.0676 (7)	
H14	0.8728	0.4205	0.0506	0.081*	
C13	0.97963 (17)	0.2836 (5)	0.08361 (19)	0.0751 (8)	
H13	1.0110	0.4074	0.0700	0.090*	
C10	0.8872 (2)	−0.0810 (5)	0.12533 (18)	0.0750 (8)	
H10	0.8562	−0.2039	0.1403	0.090*	
C15	1.14533 (12)	0.2518 (3)	0.13117 (13)	0.0779 (9)	
C16	1.20579 (14)	0.2829 (4)	0.08041 (11)	0.0981 (13)	
H16	1.2114	0.1797	0.0407	0.118*	
C17	1.25792 (12)	0.4683 (5)	0.08903 (12)	0.0986 (12)	
H17	1.2984	0.4891	0.0551	0.118*	
C18	1.24959 (12)	0.6225 (4)	0.14841 (16)	0.0937 (11)	
H18	1.2845	0.7465	0.1542	0.112*	
C19	1.18913 (14)	0.5914 (3)	0.19916 (12)	0.0873 (10)	
H19	1.1836	0.6945	0.2389	0.105*	
C20	1.13700 (11)	0.4060 (4)	0.19055 (12)	0.0786 (9)	
H20	1.0966	0.3852	0.2245	0.094*	
C11	0.9694 (2)	−0.0913 (5)	0.1338 (2)	0.0885 (10)	
H11	0.9941	−0.2238	0.1527	0.106*	
H3	0.7060 (16)	0.717 (5)	0.2355 (16)	0.070 (8)*	
O1	1.09730 (15)	0.0639 (4)	0.1246 (2)	0.1279 (16)	0.997 (9)

### Atomic displacement parameters (Å^2^)

**Table:**

	*U*^11^	*U*^22^	*U*^33^	*U*^12^	*U*^13^	*U*^23^
S1	0.0633 (5)	0.0627 (5)	0.0859 (5)	−0.0058 (3)	−0.0005 (4)	−0.0210 (4)
N3	0.0525 (11)	0.0532 (12)	0.0451 (11)	0.0006 (9)	−0.0107 (9)	−0.0013 (9)
N2	0.0555 (12)	0.0555 (12)	0.0496 (11)	0.0026 (10)	−0.0097 (9)	0.0026 (9)
N1	0.0519 (12)	0.0602 (12)	0.0485 (11)	−0.0047 (10)	−0.0077 (9)	−0.0014 (10)
C7	0.0451 (12)	0.0534 (14)	0.0431 (12)	−0.0078 (10)	−0.0040 (10)	0.0065 (10)
C6	0.0446 (12)	0.0599 (15)	0.0501 (13)	−0.0055 (11)	−0.0035 (10)	0.0106 (11)
C1	0.0546 (15)	0.0708 (18)	0.0727 (17)	0.0024 (14)	0.0016 (13)	0.0024 (15)
C2	0.0718 (19)	0.076 (2)	0.098 (2)	0.0122 (16)	0.0194 (17)	0.0087 (17)
C3	0.0581 (18)	0.104 (3)	0.104 (2)	0.0201 (18)	0.0124 (17)	0.043 (2)
C4	0.0576 (17)	0.124 (3)	0.087 (2)	0.0107 (19)	−0.0181 (16)	0.026 (2)
C5	0.0611 (17)	0.094 (2)	0.0662 (17)	−0.0001 (16)	−0.0168 (13)	0.0056 (16)
C9	0.0646 (16)	0.0480 (14)	0.0596 (15)	0.0014 (12)	−0.0005 (12)	−0.0038 (11)
C12	0.0659 (18)	0.0560 (18)	0.114 (3)	0.0176 (14)	−0.0201 (17)	−0.0137 (16)
C8	0.0505 (13)	0.0522 (14)	0.0492 (13)	−0.0070 (11)	−0.0020 (10)	0.0019 (11)
C14	0.0598 (16)	0.0539 (16)	0.088 (2)	0.0077 (13)	−0.0042 (14)	0.0132 (14)
C13	0.0616 (17)	0.0563 (17)	0.107 (2)	0.0024 (14)	−0.0034 (16)	0.0073 (16)
C10	0.087 (2)	0.0506 (16)	0.086 (2)	0.0012 (14)	−0.0052 (16)	0.0067 (14)
C15	0.0549 (16)	0.070 (2)	0.106 (2)	0.0231 (15)	−0.0157 (16)	−0.0158 (17)
C16	0.082 (2)	0.137 (4)	0.074 (2)	0.050 (2)	−0.0119 (18)	−0.029 (2)
C17	0.069 (2)	0.148 (4)	0.079 (2)	0.026 (2)	0.0060 (17)	0.027 (2)
C18	0.076 (2)	0.094 (3)	0.110 (3)	0.0044 (19)	−0.005 (2)	0.017 (2)
C19	0.081 (2)	0.085 (2)	0.096 (2)	0.0046 (18)	0.0003 (18)	−0.0168 (19)
C20	0.0653 (18)	0.083 (2)	0.088 (2)	0.0146 (16)	0.0087 (16)	−0.0074 (17)
C11	0.098 (2)	0.0485 (17)	0.116 (3)	0.0183 (17)	−0.028 (2)	0.0039 (16)
O1	0.0700 (17)	0.0667 (17)	0.242 (4)	0.0247 (12)	−0.0404 (18)	−0.0344 (18)

### Geometric parameters (Å, º)

**Table:**

S1—C9	1.765 (3)	C12—C13	1.374 (4)
S1—C8	1.740 (3)	C12—C11	1.361 (5)
N3—N2	1.362 (3)	C12—O1	1.372 (4)
N3—C7	1.334 (3)	C14—H14	0.9300
N3—H3	0.91 (3)	C14—C13	1.372 (4)
N2—C8	1.320 (3)	C13—H13	0.9300
N1—C7	1.324 (3)	C10—H10	0.9300
N1—C8	1.344 (3)	C10—C11	1.366 (5)
C7—C6	1.453 (3)	C15—C16	1.3900
C6—C1	1.3900	C15—C20	1.3900
C6—C5	1.3900	C15—O1	1.359 (3)
C1—H1	0.9300	C16—H16	0.9300
C1—C2	1.3900	C16—C17	1.3900
C2—H2	0.9300	C17—H17	0.9300
C2—C3	1.3900	C17—C18	1.3900
C3—H3A	0.9300	C18—H18	0.9300
C3—C4	1.3900	C18—C19	1.3900
C4—H4	0.9300	C19—H19	0.9300
C4—C5	1.3900	C19—C20	1.3900
C5—H5	0.9300	C20—H20	0.9300
C9—C14	1.380 (4)	C11—H11	0.9300
C9—C10	1.372 (4)		
			
C8—S1—C9	103.95 (12)	N2—C8—N1	115.2 (2)
N2—N3—H3	119.3 (18)	N1—C8—S1	119.43 (18)
C7—N3—N2	110.2 (2)	C9—C14—H14	119.7
C7—N3—H3	129.7 (18)	C13—C14—C9	120.5 (3)
C8—N2—N3	101.72 (19)	C13—C14—H14	119.7
C7—N1—C8	103.21 (19)	C12—C13—H13	120.4
N3—C7—C6	125.0 (2)	C14—C13—C12	119.3 (3)
N1—C7—N3	109.6 (2)	C14—C13—H13	120.4
N1—C7—C6	125.33 (19)	C9—C10—H10	120.0
C1—C6—C7	122.03 (14)	C11—C10—C9	120.0 (3)
C1—C6—C5	120.0	C11—C10—H10	120.0
C5—C6—C7	117.92 (14)	C16—C15—C20	120.0
C6—C1—H1	120.0	O1—C15—C16	119.5 (2)
C2—C1—C6	120.0	O1—C15—C20	120.4 (2)
C2—C1—H1	120.0	C15—C16—H16	120.0
C1—C2—H2	120.0	C15—C16—C17	120.0
C3—C2—C1	120.0	C17—C16—H16	120.0
C3—C2—H2	120.0	C16—C17—H17	120.0
C2—C3—H3A	120.0	C18—C17—C16	120.0
C2—C3—C4	120.0	C18—C17—H17	120.0
C4—C3—H3A	120.0	C17—C18—H18	120.0
C3—C4—H4	120.0	C19—C18—C17	120.0
C3—C4—C5	120.0	C19—C18—H18	120.0
C5—C4—H4	120.0	C18—C19—H19	120.0
C6—C5—H5	120.0	C18—C19—C20	120.0
C4—C5—C6	120.0	C20—C19—H19	120.0
C4—C5—H5	120.0	C15—C20—H20	120.0
C14—C9—S1	122.1 (2)	C19—C20—C15	120.0
C10—C9—S1	118.3 (2)	C19—C20—H20	120.0
C10—C9—C14	119.3 (3)	C12—C11—C10	120.6 (3)
C11—C12—C13	120.2 (3)	C12—C11—H11	119.7
C11—C12—O1	116.5 (3)	C10—C11—H11	119.7
O1—C12—C13	123.2 (3)	C15—O1—C12	119.5 (2)
N2—C8—S1	125.17 (19)		
			
S1—C9—C14—C13	−175.8 (2)	C9—C10—C11—C12	2.4 (5)
S1—C9—C10—C11	174.0 (3)	C8—S1—C9—C14	−57.9 (3)
N3—N2—C8—S1	−175.31 (18)	C8—S1—C9—C10	128.3 (2)
N3—N2—C8—N1	−0.1 (3)	C8—N1—C7—N3	0.4 (3)
N3—C7—C6—C1	12.0 (3)	C8—N1—C7—C6	−179.7 (2)
N3—C7—C6—C5	−170.67 (19)	C14—C9—C10—C11	0.0 (5)
N2—N3—C7—N1	−0.5 (3)	C13—C12—C11—C10	−2.8 (6)
N2—N3—C7—C6	179.66 (19)	C13—C12—O1—C15	24.8 (5)
N1—C7—C6—C1	−167.84 (18)	C10—C9—C14—C13	−2.0 (4)
N1—C7—C6—C5	9.5 (3)	C15—C16—C17—C18	0.0
C7—N3—N2—C8	0.4 (2)	C16—C15—C20—C19	0.0
C7—N1—C8—S1	175.31 (17)	C16—C15—O1—C12	−122.6 (3)
C7—N1—C8—N2	−0.2 (3)	C16—C17—C18—C19	0.0
C7—C6—C1—C2	177.32 (18)	C17—C18—C19—C20	0.0
C7—C6—C5—C4	−177.43 (17)	C18—C19—C20—C15	0.0
C6—C1—C2—C3	0.0	C20—C15—C16—C17	0.0
C1—C6—C5—C4	0.0	C20—C15—O1—C12	60.7 (4)
C1—C2—C3—C4	0.0	C11—C12—C13—C14	0.8 (5)
C2—C3—C4—C5	0.0	C11—C12—O1—C15	−158.7 (3)
C3—C4—C5—C6	0.0	O1—C12—C13—C14	177.2 (3)
C5—C6—C1—C2	0.0	O1—C12—C11—C10	−179.5 (3)
C9—S1—C8—N2	−34.3 (2)	O1—C15—C16—C17	−176.7 (2)
C9—S1—C8—N1	150.7 (2)	O1—C15—C20—C19	176.7 (2)
C9—C14—C13—C12	1.6 (5)		

### Hydrogen-bond geometry (Å, º)

**Table:**

*D*—H···*A*	*D*—H	H···*A*	*D*···*A*	*D*—H···*A*
N3—H3···N2^i^	0.91 (3)	2.05 (3)	2.944 (3)	170 (2)
C16—H16···S1^ii^	0.93	2.77	3.694 (2)	170

Symmetry codes: (i) −*x*+3/2, *y*+1/2, −*z*+1/2; (ii) −*x*+2, −*y*, −*z*.
